# Effects of Different Forms of Organic Selenium on Growth Performance, Antioxidant Capacity, and Intestinal Health in Rice Field Eel (*Monopterus albus*)

**DOI:** 10.3390/ani15131949

**Published:** 2025-07-02

**Authors:** Denghang Yu, Yujia Liu, Jincheng Wan, Jiaxiang Chen, Yangjie Qiu, Chi Zhang

**Affiliations:** Hubei Key Laboratory of Animal Nutrition and Feed Science, School of Animal Science and Nutritional Engineering, Wuhan Polytechnic University, Wuhan 430023, China; yudenghang1985@163.com (D.Y.); 18365970228@163.com (Y.L.); m13329798888@163.com (J.W.); chenjiaxiang777@163.com (J.C.); 15286303323@163.com (Y.Q.)

**Keywords:** *Monopterus albus*, organic selenium, growth performance, antioxidant capacity, gut microbiota

## Abstract

To evaluate the effects of different organic selenium sources on rice field eel (*Monopterus albus*), four isonitrogenous and isolipidic diets were tested: a control (no supplement) and four diets each supplemented with 0.24 mg/kg selenium as diselenoacetic acid (Se1), diselenopropionic acid (Se2), diselenobutyric acid (Se3), or diselenodibutyric acid (Se4). Rice field eels (initial body weight 18.43 ± 0.03 g) were fed for 60 days. The Se1 group showed significantly higher WGR and SGR than the control. Whole-body crude protein was significantly increased in the Se3 and Se4 groups, while crude lipid content was significantly decreased in Se1, Se3, and Se4 groups. Selenium concentrations in the liver and kidney were highest in the Se2 group. Serum AST and ALT activities were decreased in Se4. In the Se4 group, intestinal CAT, T-SOD, and GSH-PX activities were enhanced, along with upregulation of Gpx8, CAT, Nrf2, and Keap1. Hepatic CAT and GSH-PX increased in Se2 and Se4, with Gpx8 upregulated in Se1. Intestinal lipase activity was significantly increased in Se2. At the phylum level, *Proteobacteria* predominated. At the genus level, *Cetobacterium* predominated. In summary, diselenoacetic acid enhanced growth, while diselenopropionic acid and diselenodibutyric acid improved antioxidant defense and intestinal health in rice field eel.

## Simple Summary

This study systematically evaluated the effects of four organic selenium compounds—diselenoacetic acid, diselenopropionic acid, diselenobutyric acid, and diselenodibutyric acid—on growth performance, antioxidant capacity, and intestinal health in rice field eel. Rice field eels were fed isonitrogenous and isolipidic diets supplemented with one of these organic selenium sources or no selenium (control) for 60 days. The results showed that diselenoacetic acid significantly increased weight gain and specific growth rate, while diselenobutyric acid enhanced hepatic and intestinal antioxidant enzyme activities. Supplementation with diselenopropionic acid notably improved intestinal digestive enzyme activity, preserved villus structure, and promoted the abundance of beneficial gut bacteria, thereby supporting nutrient absorption. These findings demonstrate that targeted organic selenium supplementation can effectively improve growth, antioxidant defense, and gut health in rice field eel. The study provides new insights into functional feed development and offers practical guidelines for optimizing selenium nutrition in intensive aquaculture, with the goal of reducing oxidative stress and mortality in rice field eel production.

## 1. Introduction

Selenium (Se), an essential trace element in animals, plays a crucial role in maintaining normal physiological functions. In intensive aquaculture systems, selenium is predominantly absorbed by fish via feed additives, where it exists in two main forms: inorganic selenium (e.g., sodium selenite) and organic selenium (e.g., selenomethionine and selenium-enriched yeast). Studies on grass carp (*Cenopharyngodon idellus*) [1] and Wuchang bream (*Megalobrama amblycephala*) [2] have demonstrated that the incorporation of an appropriate amount of sodium selenite significantly enhances the weight gain rate, specific growth rate, and antioxidant enzyme activity in fish. The supplementation of yeast selenium and selenomethionine in the feed of the Atlantic croaker (*Micropogonias undulatus*) [3] and the Siberian sturgeon (*Acipenser baerii*) [4], respectively, can promote fish growth and enhance antioxidant capacity. Numerous studies have demonstrated the superior efficacy of organic selenium, which exhibits higher bioavailability, lower toxicity, reduced environmental impact, and greater assimilation efficiency compared to inorganic forms [5]. At the molecular level, selenium’s physiological functions are mediated through two key mechanisms. Regarding growth regulation, selenium is incorporated as selenocysteine into the catalytic center of thyroxine deiodinase (DIO), a critical enzyme regulating fish growth and development [6]. Dietary selenium supplementation has been shown to enhance growth performance in grass carp by modulating the growth hormone–insulin-like growth factor axis and hypothalamic–pituitary–thyroid axis [7]. Supplementation with 4 mg/kg selenium yeast significantly improved weight gain and specific growth rate in rainbow trout [8]. Additionally, digestive enzyme activities are enhanced by selenium supplementation, thereby directly promoting growth in aquatic animals. Studies have shown that selenium supplementation significantly increases digestive enzyme activities, including those of amylase and lipase, in grass carp, which ultimately stimulates growth [9]. Within the antioxidant defense system, selenium is an essential component of major antioxidant enzymes, including glutathione peroxidase (GSH-PX) and thioredoxin reductase (TRXR). Reducing cellular concentrations of hydrogen peroxide (H_2_O_2_) and organic hydroperoxides helps to mitigate potential free radical damage and prevent lipid peroxidation [10]. Appropriate supplementation with organic selenium has been shown to significantly increase the activities of total superoxide dismutase (T-SOD) and GSH-PX enzymes in *Haliotis discus hannai* [11].

The threshold between optimal and toxic selenium levels in animals is extremely narrow. The National Research Council reports the dietary selenium requirement for fish is approximately 0.1–0.5 mg/kg [12]. Wang et al. [13] documented that selenium requirements among major farmed fish species range from 0.09 mg/kg in channel catfish to 20.26 mg/kg in Chinese sturgeon. Extensive research demonstrates that selenium-deficient diets result in stunted growth, reduced feed efficiency, and impaired antioxidant and immune functions in fish. Conversely, excessive selenium induces toxicity, causing oxidative stress, lipid metabolism disorders, tissue damage, and other adverse effects. Optimal supplementation with organic selenium enhances growth performance and non-specific immunity in various aquaculture species including grass carp [14] and gibel carp (*Carassius auratus gibelio*) [15]. Thus, when administered as a feed additive at appropriate concentrations, organic selenium substantially improves aquatic animal health.

Rice field eel (*Monopterus albus*) is considered a premium freshwater aquaculture species due to its high protein and low lipid content [16]. In 2023, rice field eel production reached 355,200 metric tons, reflecting a 6.28% year-on-year increase compared to 2022. Responding to increasing market demand, the industry has shifted to large-scale, intensive farming methods. However, high-density cultivation environments frequently result in stunted growth, compromised immunity, and increased oxidative stress in rice field eel, thereby heightening susceptibility to disease and elevating mortality rates. Previous studies have shown that organic selenium offers greater advantages compared to inorganic selenium [5]. Currently, our laboratory is focusing on the study of several different forms of selenium, while also aiming to investigate the specific effects of these various selenium forms on the growth of rice field eels. This study investigates the effects of four distinct organic selenium compounds—diselenoacetic acid, diselenopropionic acid, diselenobutyric acid, and diselenodibutyric acid—used as dietary supplements on farmed rice field eel. The research evaluates the effects of these compounds on growth performance, blood biochemical parameters, antioxidant activity, and gut health, aiming to mitigate oxidative stress in aquaculture. By identifying the most effective organic selenium formulation, this study provides both theoretical foundations and practical guidelines for optimizing selenium supplementation in rice field eel diets.

## 2. Materials and Methods

### 2.1. Experimental Design and Feed Preparation

Healthy male adult rice field eels (initial body weight of 18.43 ± 0.03 g) were randomly assigned to five groups (one control, four treatments), each with three replicates of 30 eels housed in 1.5 × 2 m net cages. Experimental diets differed only in selenium source: the control (Se0) contained no selenium, while Se1–Se4 had 0.24 mg/kg selenium from diselenoacetic acid, diselenopropionic acid, diselenobutyric acid, and diselenodibutyric acid. The recommended added dose is based on Gong Bing’s research [17], which indicates that the optimal amount of selenium atoms in selenomethionine is 0.24 mg/kg. The trial lasted 60 days. Feed composition and nutrient levels appear in Table 1. The basal diet included fish meal (protein), wheat flour (carbohydrate), and a 1:1 mix of fish oil and soybean lecithin (lipid). Other ingredients were sourced from Wuhan CP Aquaculture Co., Ltd. All were ground, sieved (40-mesh), weighed, blended stepwise, then pelleted (2.0 mm), air-dried, labeled, and stored at −5 °C.

### 2.2. Aquaculture Management

The rice field eels were sourced from Hubei Xiantao Rice Field Eel Industry Group Co., Ltd. in Xiantao, China and were acclimated for one week at the Xiantao Rice Field Eel Industry Technology Research Institute. Water hyacinth, provided by the same company, can purify water, provide shade and reduce temperature, alleviate high-temperature stress, offer hiding spaces for rice field eels, and reduce mutual fighting. Culture water came from filtered local wells with controlled parameters: temperature 25–30 °C, pH 6.50–7.50, ammonia ≤ 0.15 mg/L, nitrite ≤ 0.05 mg/L, and dissolved oxygen > 6 mg/L, meeting Chinese fishery standards. Fish were fed once daily (18:00) following the “four fixed principles.” Uneaten feed was removed at 06:00. Water was managed according to conditions. Pest control was applied to water hyacinth as needed.

### 2.3. Sample Collection

After 24 h of fasting, rice field eels were anesthetized with 30 mg/L MS-222 (Tricaine methanesulfonate, M14788) from AbMole in Shanghai, China, prior to sampling. The eels in each culture tank were counted and weighed to calculate final body weight (FBW), weight gain ratio (WGR), specific growth ratio (SGR), and feed conversion ratio (FCR). In each culture tank, five rice field eels were randomly selected to weigh and measure for length. Following tail amputation for blood sampling, eels were dissected on ice, with visceral and liver weights measured for morphometrics. Liver and intestinal tissues were flash-frozen in liquid nitrogen and stored at −80 °C. Blood was left at 4 °C/24 h; serum was collected and stored at −80 °C for analysis. In each culture tank, three rice field eels were randomly selected for whole-body nutritional analysis.

### 2.4. Calculation of Growth Performance and Morphological Parameters

Specific growth rate (SGR, %/d) = (LnW_t_ − LnW_0_)/t × 100, (1)

Feed conversion ratio (FCR) = F/(W_t_ − W_0_), (2)

Viscerasomatic index (VSI, %) = (VW/W) × 100,(3)

Hepatosomatic index (HSI, %) = (HW/W) × 100,(4)

Condition factor (CF, g/cm^3^) = W/L^3^, (5)

Weight gain ratio (WGR, %) = (W_t_ − W_0_)/W_0_ × 100 (6)

In these formulas: W_0_ and W_t_ are the initial and final average weights (g) of the eels; t is the culture duration (days); F is the average feed intake (g); VW, HW, and W represent visceral, liver, and whole-body weights (g), respectively; and L is body length (cm).

### 2.5. Determination of Nutritional Components

Nutritional components in feed and fish samples were analyzed using standard protocols: moisture (GB/T 6435-2014) [18], crude protein (GB/T 6432-2018) [19], crude fat (GB/T 6433-2006) [20], and crude ash (GB/T 6438-2007) [21].

### 2.6. Determination of Selenium Content

For selenium determination, feed samples were crushed, sieved (1 mm), and stored at room temperature; viscera samples were dried at 65 °C for 48 h, ground, and kept at −20 °C. All samples were digested using a microwave system (TANK40, Xinyi Microwave Chemistry, Shanghai, China).

### 2.7. Liver and Intestinal Biochemical Indices

After the feeding trial, rice field eels fasted for 24 h. Three eels per cage were randomly selected, and blood collected from the caudal vein for serum albumin (ALB, A028-2-1), total protein (TP, A045-2-2), total cholesterol (TC, A111-1-1), triglyceride (TG, A110-1-1), glucose (GLU, F006-1-1), high-density lipoprotein (HDL, A112-1-1) or low-density lipoprotein (LDL, A113-1-1), aspartate aminotransferase (AST, C010-2-1), and alanine aminotransferase (ALT, C009-2-1) analyses. Liver and intestine were dissected to assay catalase (CAT, A007-1-1), glutathione peroxidase (GSH-PX, A006-2-1), superoxide dismutase (SOD, A001-3-2), malondialdehyde (MDA, A003-1-2), trypsin (A080-2-2), amylase (AMS, C016-1-2), and lipase (LPS, A054-2-1) activities using kits from Nanjing Jiancheng Bioengineering Institute (Nanjing, China).

### 2.8. Measurement of Related Antioxidant Gene Expression Levels

The published genome sequence information of rice field eels was obtained from NCBI, leading to the selection of RPL17 as the reference gene. Primers were then designed using Primer 5.0 software and synthesized by Qingke Biotechnology Co., Ltd. (Beijing, China). The gene sequences are shown in Table 2. The RNA was extracted using RNAiso Easy kit (9109) Reverse transcription was conducted to synthesize complementary DNA (cDNA) following the protocol outlined in the PrimeScript RT Reagent Kit with gDNA Eraser (Perfect Real Time) (RR047A). Real-time quantitative PCR (qPCR) was conducted using a Roche LIghtCycler 480 system and TB Green^®^ Premix Ex Taq II kit (RR820A), following the manufacturer’s instructions. Cycling conditions were 95 °C for 30 s (pre-denaturation), then 40 cycles of 95 °C for 5 s and 60 °C for 30 s. Relative gene expression was calculated using the 2^−ΔΔCt^ method [22]. All the kits were from TaKaRa Biotechnology Co., Ltd. in Shanghai, China.

### 2.9. Analysis of Intestinal Tissue Structure

Intestinal tissues were fixed in 4% paraformaldehyde for at least 48 h, then sent to Servicebio Company for tissue sectioning. Samples were dehydrated through graded ethanol and xylene. Samples were embedded in paraffin and sectioned at 4 μm thickness using a microtome. Sections were stained with hematoxylin and eosin (H&E) and examined under an optical microscope (Olympus BX53, Tokyo, Japan). Villus width (VW), villus height (VH), and muscle layer thickness (MT) were measured using ImageJ software (2019).

### 2.10. Analysis of Intestinal Microbiota

After the culture period, rice field eels were fed to satiation. Twenty-four hours later, six eels from each cage in all five groups were randomly selected for dissection. Posterior intestinal contents were collected into sterile 2 mL tubes and stored at −80 °C for subsequent microbiota analysis. Sequencing was performed by Shanghai Majorbio Bio-pharm Technology Co., Ltd. (Shanghai, China). The sequencing experimental procedure involved thawing the samples at −80 °C; cutting 50 mg of the sample into a homogenization tube; adding 725 μL of SLX-Mlus buffer (PD090) for centrifugation; and extracting the total intestinal DNA from the supernatant. The 16S rRNA high-throughput sequencing (Illumina MiSeq PE300 platform, San Diego, CA, USA) was selected, and the obtained sequence data were uploaded to the NCBI SRA database.

### 2.11. Data Processing and Analysis

The scheme of all animals and experimental timelines appears in Table 3. The data were presented as mean ± standard deviation (S.D., *n* = 3). One-way ANOVA was performed using SPSS 26.0 (SPSS, Chicago, IL, USA), and Duncan’s method was used for multiple comparisons. Differences were considered significant at *p* < 0.05.

## 3. Results

### 3.1. Growth Performance

As shown in Table 4, there were no significant differences in feed conversion ratios among the groups (*p* > 0.05). Compared with the control and other experimental groups, the Se1 group exhibited significantly higher FW, WGR, and SGR in rice field eels (*p* < 0.05), indicating that dietary supplementation with 0.42 mg/kg diselenoacetic acid could promote the growth of rice field eels.

### 3.2. Morphological Parameters

As shown in Table 5, dietary supplementation with different forms of organic selenium did not significantly affect CF, HSI, or VSI of rice field eel (*p* > 0.05). Nevertheless, the Se2 group exhibited higher VSI and HSI than other treatment groups.

### 3.3. Whole-Body Composition

As shown in Table 6, the Se3 group exhibited significantly higher crude protein content in rice field eel compared to both the control and other experimental groups, whereas the Se1 and Se2 groups showed significantly lower levels (*p* < 0.05). Relative to the control, crude lipid content was significantly reduced in the Se1, Se3, and Se4 groups (*p* < 0.05), but significantly elevated in the Se2 group (*p* < 0.05). The crude ash content in the Se3 and Se4 groups was significantly greater than that of the control group (*p* < 0.05).

### 3.4. Se Accumulation

**Figure 1:**
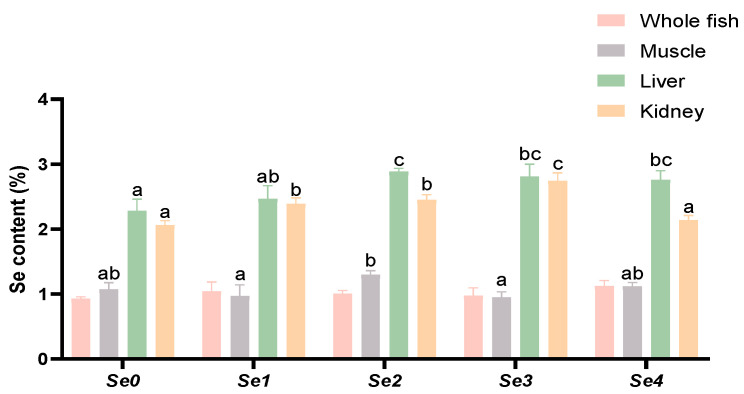
Effects of different forms of organic selenium on Se accumulation in rice field eel tissues. The data were shown as mean ± standard error (*n* = 3), with different letters indicating significant differences among different groups (*p* < 0.05).

As shown in Figure 1, dietary supplementation with different forms of organic selenium did not significantly affect the whole-body selenium content of rice field eels (*p* > 0.05). Regarding muscle selenium content, the Se2 group exhibited significantly higher levels than both Se1 and Se3 groups (*p* < 0.05), while no significant differences were observed among the other experimental groups (*p* > 0.05). For hepatic selenium content, the Se2, Se3, and Se4 groups all showed significantly higher values than the control group (*p* < 0.05), with the Se2 group being significantly higher than Se1. In renal selenium content, no significant difference was found between the Se4 group and the control (*p* > 0.05), while all other experimental groups demonstrated significantly higher levels than the control (*p* < 0.05). Notably, the Se3 group showed significantly higher selenium content than the other experimental groups (*p* < 0.05).

### 3.5. Serum Biochemical Parameters

As shown in Table 7, no significant differences were observed in TP, ALB, TG, HDL, or LDL levels among the groups (*p* > 0.05). Compared with the control group, serum AST levels were significantly higher in the Se3 group (*p* < 0.05), whereas the Se4 group had significantly lower AST and ALT levels (*p* < 0.05). The Se2 group showed a significant increase in serum TC concentration (*p* < 0.05). Relative to the other experimental groups, the Se1 group had significantly higher serum GLU levels (*p* < 0.05).

### 3.6. Hepatic and Intestinal Antioxidant Parameters

**Figure 2:**
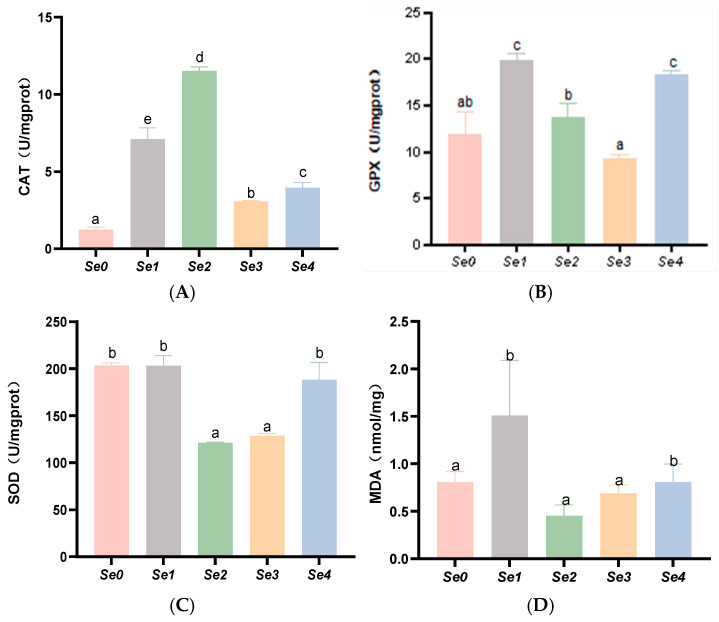
Effects of different forms of organic selenium on antioxidant parameters in the liver in rice field eel. (**A**) CAT; (**B**) GPX; (**C**) SOD; and (**D**) MDA, with different letters indicating significant differences among different groups (*p* < 0.05).

As shown in Figure 2, the experimental groups exhibited significantly higher hepatic CAT activity than the control group (*p* < 0.05), with peak activity observed in the Se2 group. The Se1 and Se4 groups showed markedly elevated hepatic GSH-PX (GPX) activity compared to controls (*p* < 0.05). Notably, hepatic malondialdehyde (MDA) content was significantly increased in the Se1 and Se4 groups relative to both the control and other experimental groups (*p* < 0.05). No significant difference was found in hepatic T-SOD activity between the Se1 and Se4 groups and controls (*p* > 0.05).

**Figure 3:**
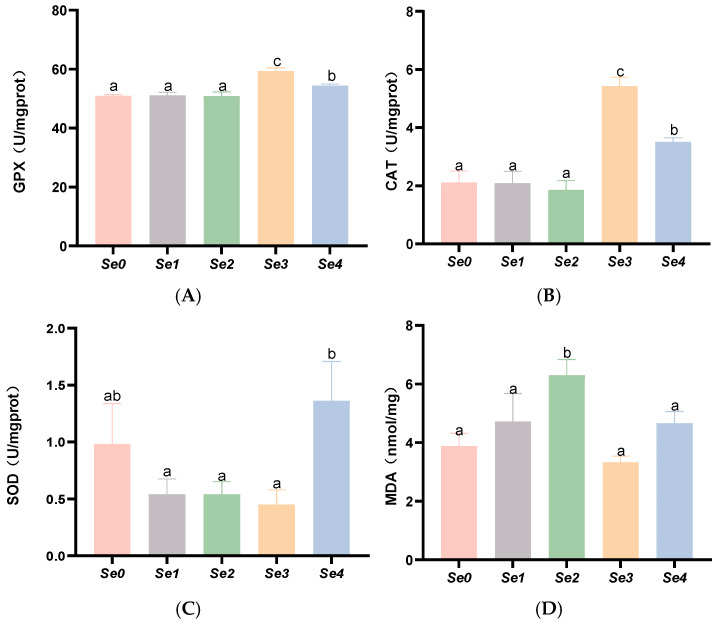
Effects of different forms of organic selenium on antioxidant parameters in the intestine in rice field eel. (**A**) GPX; (**B**) CAT; (**C**) SOD; and (**D**) MDA, with different letters indicating significant differences among different groups (*p* < 0.05).

As shown in Figure 3, intestinal CAT and GPX activities were significantly higher in the Se3 and Se4 groups than in the control group (*p* < 0.05). Compared with the control group and other experimental groups, the MDA content in the intestines of the Se2 group was significantly increased (*p* < 0.05). Compared with the other experimental groups, T-SOD levels in the intestines of the Se2 and Se4 groups were significantly elevated (*p* < 0.05), while no significant difference was observed between the control group and the experimental groups (*p* > 0.05).

### 3.7. Expression Levels of Antioxidant-Related Genes in the Liver and Intestine

**Figure 4:**
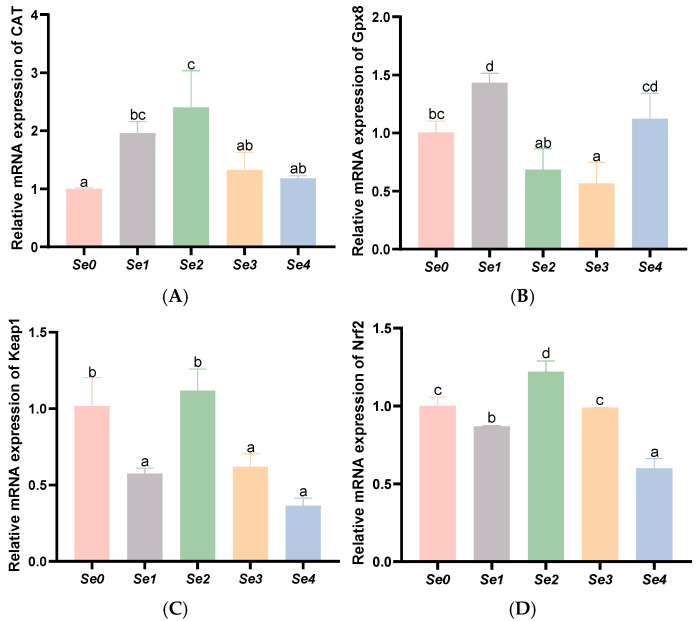
Effects of different forms of organic selenium on the expression of antioxidant genes in the liver in rice field eel. (**A**) *CAT*; (**B**) *Gpx8*; (**C**) *Keap1*; and (**D**) *Nrf2*, with different letters indicating significant differences among different groups (*p* < 0.05).

As shown in Figure 4, hepatic *Gpx8* expression was significantly elevated in the Se1 group compared to both the control and other experimental groups (*p* < 0.05). Similarly, hepatic *CAT* expression showed significant increases in both Se1 and Se2 groups (*p* < 0.05), while liver *Nrf2* and *Keap1* levels were markedly higher in the Se2 group (*p* < 0.05).

**Figure 5:**
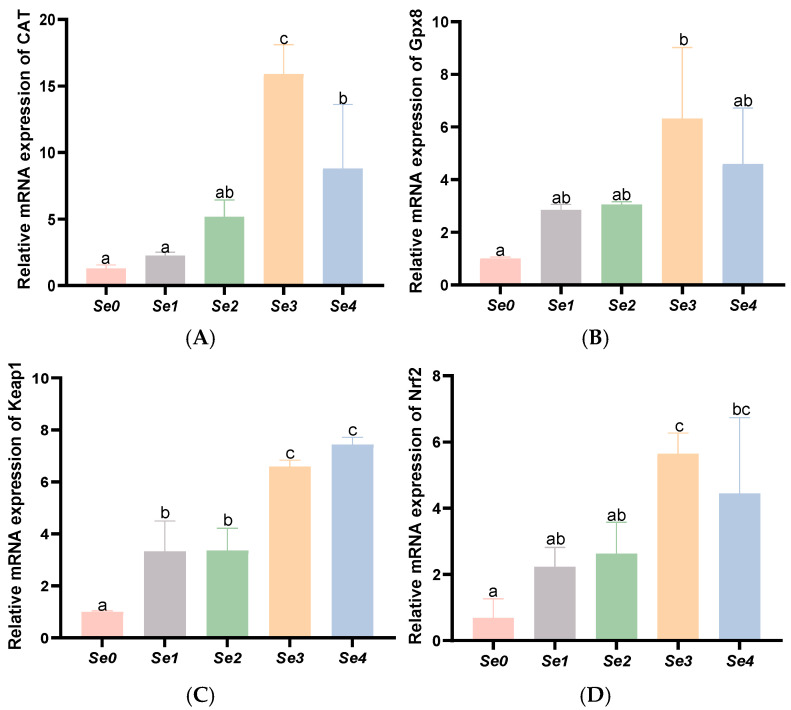
Effects of different forms of organic selenium on in the expression of antioxidant genes in the intestine in rice field eel. (**A**) *CAT*; (**B**) *Gpx8*; (**C**) *Keap1*; and (**D**) *Nrf2*, with different letters indicating significant differences among different groups (*p* < 0.05).

As shown in Figure 5, intestinal *Gpx8*, *CAT*, *Nrf2*, and *Keap1* expression levels were significantly upregulated in the Se3 and Se4 groups compared to controls and other experimental groups (*p* < 0.05).

### 3.8. Intestinal Histomorphology

**Figure 6:**
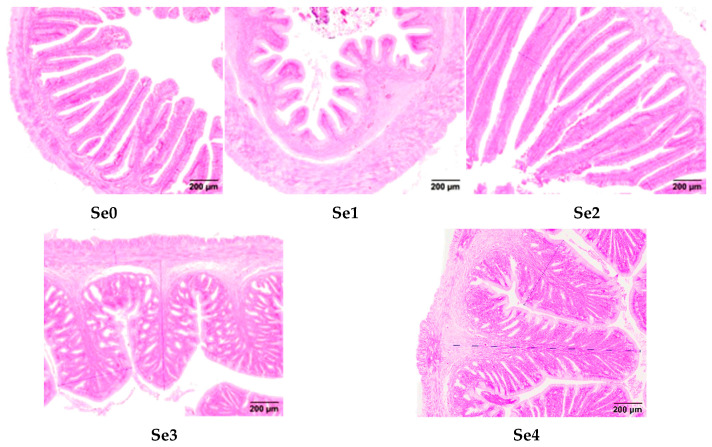
Effects of different forms of organic selenium on the morphological structure in rice field eel intestinal tissue (100×).

**Figure 7:**
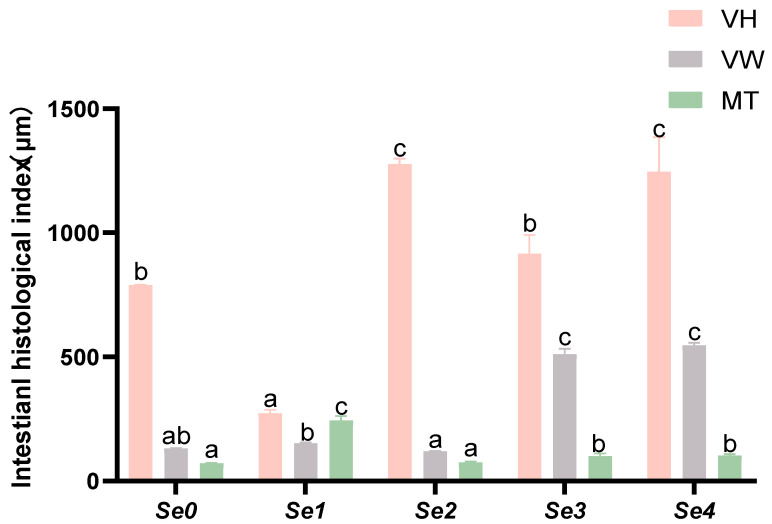
Effects of different forms of organic selenium on the morphological indices of the intestinal tissue in rice field eel, with different letters indicating significant differences among different groups (*p* < 0.05).

Figure 6 illustrates the morphological alterations in transverse midgut sections of rice field eels fed different forms of organic selenium. The Se2 group displayed well preserved intestinal villi structure, characterized by slender, finger-shaped projections with optimal morphology. Figure 7 demonstrates the intestinal histomorphological parameters, revealing significantly greater VH in Se2 and Se4 groups compared to controls (*p* < 0.05), whereas Se3 showed no significant difference (*p* > 0.05). VW was significantly increased in Se3 and Se4 groups (*p* < 0.05). MT was significantly enhanced in Se1, Se3, and Se4 groups (*p* < 0.05), with Se2 exhibiting no significant difference from controls (*p* > 0.05).

### 3.9. Intestinal Digestive Enzyme Activities

**Figure 8:**
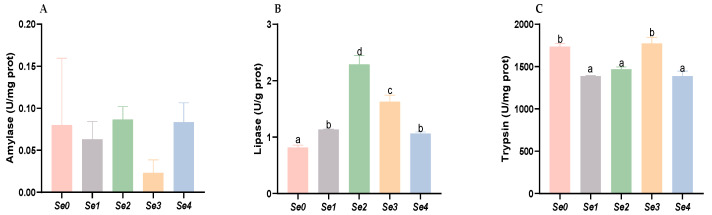
Effects of different forms of organic selenium on the digestive enzyme activity of the intestine in rice field eel. (**A**) Amylase; (**B**) lipase; and (**C**) trypsin, with different letters indicating significant differences among different groups (*p* < 0.05).

As shown in Figure 8, intestinal lipase activity was significantly higher in the experimental groups compared to the control, with the Se2 group demonstrating the most pronounced enhancement (*p* < 0.05). Trypsin activity showed significant reduction in the Se1, Se2, and Se4 groups (*p* < 0.05). No statistically significant difference was observed in intestinal amylase activity between experimental and control groups (*p* > 0.05).

### 3.10. Intestinal Microbial Flora

As shown in Table 8, supplementation with different forms of organic selenium did not significantly affect the ACE, Chao, Shannon, and Simpson index of rice field eel (*p* > 0.05).

**Figure 9:**
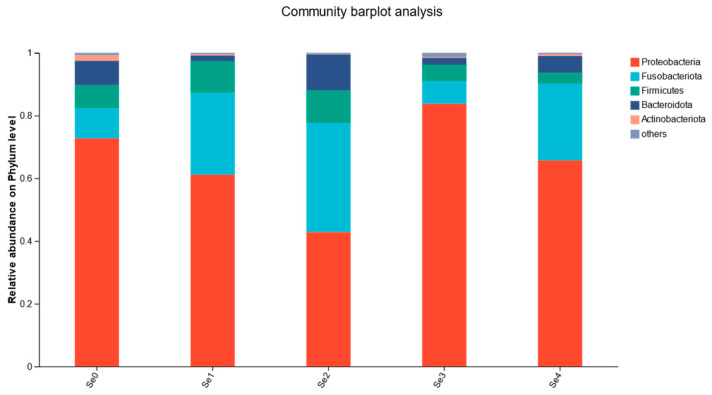
Effects of different forms of organic selenium on the composition of intestinal flora in rice field eel (phylum level).

**Figure 10:**
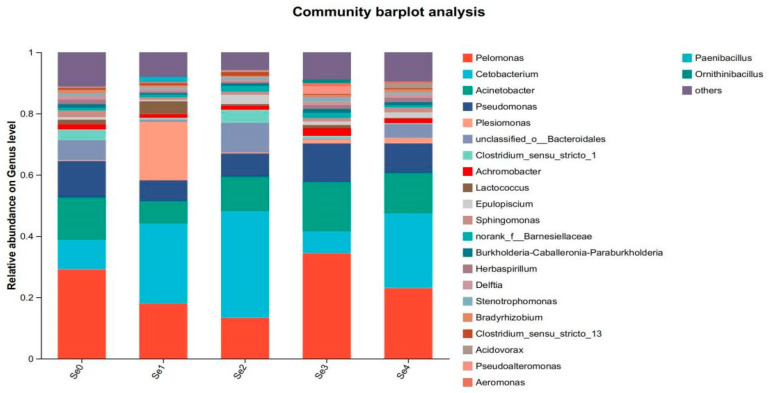
Effects of different forms of organic selenium on the composition of intestinal flora in rice field eel (genus level).

As shown in Figure 9, the dominant phyla at the phylum level included *Proteobacteria*, *Fusobacteria*, *Firmicutes*, *Bacteroidetes*, and *Actinobacteria*, with *Proteobacteria*, *Fusobacteria*, and *Bacteroidetes* being the predominant bacteria. As shown in Figure 10, the dominant populations at the genus level included *Pelomonas*, *Cetobacterium*, *Acinetobacter*, *Pseudomonas*, and *Plesiomonas*.

## 4. Discussion

### 4.1. Growth Performance

Selenium, as an essential trace element, plays a crucial role in the growth and development of fish. In recent years, selenium has increasingly been incorporated as a nutritional additive in fish feed. In this experiment, the addition of 0.42 mg/kg diselenoacetic acid significantly enhanced the SGR and WGR of rice field eel, while no significant differences were observed between other experimental groups and the control group. This finding is consistent with the effects reported for selenomethionine supplementation in rice field eel. Numerous studies have demonstrated that appropriate dietary selenium supplementation can promote fish growth. For example, supplementing feed with a suitable concentration of selenium significantly enhanced the growth and feed conversion rate of Nile tilapia [23]; Marwa et al. reported that European sea bass fed with 0.5 mg/kg nano-selenium exhibited significantly higher FBW, WG, and SGR compared to the control group [24]. Similarly, Dawood et al. found that supplementing red sea bream diets with 1.0 mg/kg nano-selenium significantly increased FBW, WG, and SGR [25]. In this experiment, diselenoacetic acid promoted growth, while other selenium sources showed no significant effects on the growth of rice field eel. This lack of effect may be attributed to the supplementation levels of these selenium sources being neither deficient nor excessive, resulting in neither growth promotion nor inhibition in the short term. Differences in their biological efficacy were mainly reflected in tissue accumulation rather than growth. However, the specific mechanisms underlying these differences warrant further investigation.

### 4.2. Whole-Body Composition and Morphological Indices

The evaluation of fish nutritional value primarily depends on the moisture, protein, and lipid content in the muscle. In this experiment, the crude protein content significantly increased with the addition of diselenobutyric acid to the basal diet, indicating that selenium supplementation in feed enhances the protein content in fish meat, thereby improving its nutritional value. Dawood et al. found that dietary supplementation with selenium nanoparticles in red sea bream feed increased the crude protein content of the whole fish at different concentrations, with significant differences compared to the control group [25]. These findings are largely consistent with the results of this study, where the crude protein content in fish significantly decreased with the addition of diselenoacetic acid and diselenopropionic acid, while diselenodibutyric acid showed no significant effect, suggesting that different organic selenium compounds may have varying impacts on the crude protein content in fish at the same concentration. Except for diselenopropionic acid, which had no significant effect, the crude lipid content in fish significantly decreased with other selenium sources. Similarly, Liu et al. reported that dietary selenium supplementation significantly reduced crude lipid content in grass carp, with exosomal miR-22 implicated in the selenium-mediated attenuation of hepatic lipid accumulation [5]. These results align with the findings of this experiment, indicating that appropriate selenium supplementation in feed can influence muscle fat metabolism in fish to some extent. The crude ash content in the diselenobutyric acid and diselenodibutyric acid experimental groups was significantly higher than in the control group, reflecting that selenium can be deposited in fish muscle to some degree.

CF, HSI, and VSI are morphological indices used to assess the energy and nutritional status of fish [26]. After 60 days of the feeding trial in this study, there were no significant differences in CF, VSI, and HSI between the experimental groups and the control group of rice field eel, indicating that the addition of different selenium forms to the feed did not affect the morphological indices in this experiment. This finding aligns with some previous studies [27,28], and it is speculated that variations in feed formulation, cultured species, and rearing environments may contribute to the differing results.

### 4.3. Se Accumulation

Compared to inorganic selenium, organic selenium is more readily absorbed and converted by the organism, accumulating in tissues. In this study, dietary selenium supplementation did not significantly affect selenium concentrations in the whole fish or muscle tissues of rice field eels, but it did significantly increase selenium levels in the liver and kidneys. This finding may be attributed to the liver being the first organ to encounter selenium post-absorption, as well as its central role in selenoprotein synthesis and selenium metabolism. After ingestion, selenium—primarily present as selenomethionine—is converted into selenocysteine, and through β-lyase activity, generates H_2_Se, which participates in selenoprotein synthesis [29]. The remaining selenium is primarily excreted through urine produced by the kidneys and feces, leading to a significantly higher accumulation of selenium in the kidneys compared to other tissues. Studies on groupers have shown that, compared to inorganic selenium, organic selenium is more easily deposited in muscles. At a dietary selenium level of 1.5 mg/kg, the muscle selenium concentration in fish fed organic selenium was more than three times higher than in those fed inorganic selenium [30]. The findings differ from the present study, which may be due to species-specific metabolism; as carnivorous fish, rice field eels may rely more on liver processing and kidney excretion of selenium rather than muscle storage.

### 4.4. Serum Biochemical Parameters

Serum biochemical indicators are widely used to assess the overall health status of fish in physiological processes [31]. Serum TP and ALB play important roles in immune response mechanisms [32]. In this experiment, supplementation with different forms of organic selenium had no significant effect on the plasma TP and ALB levels in rice field eels. Dawood et al. reported that dietary supplementation with 1 mg/kg nano-selenium significantly increased serum total protein content in Nile tilapia but had no significant effect on serum ALB [23]. Mansour et al. found that dietary supplementation with 2.97–3.98 mg/kg yeast selenium significantly increased serum TP and ALB levels in *Argyrosomus regius* [32]. These results differ from those of the present study, which may be attributed to differences in species, environmental conditions, or the relatively low concentration of organic selenium added.

Serum AST and ALT activities are important indicators of liver health. In this experiment, dietary supplementation with diselenodibutyric acid significantly reduced AST and ALT activities, indicating improved liver function in rice field eels. Soliman et al. reported that nano-selenium particles reduced AST and ALT activities, thereby enhancing the liver health of catfish [33]. These findings are consistent with the present findings. However, dietary supplementation with 0.3–0.9 mg/kg organic selenium had no significant effect on plasma AST and ALT activities in *Carassius auratus* [34], which may be attributed to differences in selenium dosage and its varying effects on the health status of tissues and organs.

The levels of GLU, TC, and TG serum reflect the body’s lipid metabolism capacity [32]. In this experiment, TG and GLU showed no significant difference compared to the control group, while the addition of selenopropionic acid increased TC levels. No significant effects were observed in the other treatment groups compared to the control. Deilamy Pour et al. found that nano-selenium supplementation in cobia diets reduced serum GLU, TC, and TG levels [35], which is inconsistent with the results of this study. The discrepancy may be attributed to the carnivorous nature of rice field eels, which rely primarily on protein and fat for energy metabolism and have limited capacity for carbohydrate utilization, leading to differences in GLU regulation mechanisms. Additionally, the high fat content of the feed in this experiment might have masked the effect of selenium on TG.

### 4.5. Hepatic and Intestinal Antioxidant Capacity

Excessive production of reactive oxygen species can damage the lipid content in cell membranes, induce lipid peroxidation, and impair RNA, which can be assessed by the level of MDA [36]. Selenium, as a functional component of GSH-PX, is incorporated as selenocysteine, serving as an active site within the enzyme. It plays a crucial role in activating the antioxidant defense system, with its antioxidant activity being the most important biological function of selenium. Antioxidant enzymes (CAT, SOD, T-AOC, and GSH-PX) constitute the first line of defense in the antioxidant system of fish [37]. In this study, supplementation with diselenodibutyric acid significantly increased the activities of GSH-PX and CAT in both the intestine and liver, potentially due to enhanced selenium deposition in the liver. Supplementation with different forms of organic selenium enhanced hepatic CAT activity, while diselenoacetic acid and diselenodibutyric acid increased GSH-PX activity. Zhang et al. demonstrated that dietary supplementation with 0.44–0.77 mg/kg selenomethionine in striped sea bass increased liver GSH-PX, T-SOD, and CAT activities in a selenium dose-dependent manner, reaching peak levels [38]. This result is consistent with the findings of the present study. However, in this experiment, supplementation with different forms of organic selenium had no significant effect on MDA levels or T-SOD activity. This may be due to variations in the effective doses of different selenium forms in organisms, and the dosage of organic selenium in this experiment did not reach the threshold required to significantly influence MDA or T-SOD. Differences in the bioavailability of various selenium forms or the organic selenium dosage in this study may not have reached the threshold necessary to significantly affect MDA and T-SOD.

Antioxidant enzyme activity in animals is partly regulated at the gene expression level corresponding to each enzyme. This finding aligns with some previous studies on selenium forms and the upregulated gene expression levels of Gpx8 and CAT, which were largely consistent with the observed enzyme activity trends. Yu et al. reported that hepatic GSH-PX and CAT gene expression in grass carp fed 0.6–0.9 mg/kg nano-selenium was significantly increased [39], which is consistent with the findings of this experiment.

Nuclear factor erythroid 2-related factor 2 (Nrf2) is a key regulator of cellular redox balance and plays an anti-inflammatory role by protecting antioxidants involved in growth factor signaling, nutrient balance, and protein folding [40]. Under oxidative stress, Nrf2 translocates into the nucleus and initiates the transcription of antioxidant genes responsible for ROS clearance [41]. However, the accumulation of Nrf2 in the nucleus can lead to free radical damage, apoptosis, and tumorigenesis. In such cases, Kelch-like ECH-associated protein 1 (Keap1) acts as a suppressor to shut down the Nrf2 response [42]. The results of this experiment showed that dietary supplementation with selenium significantly upregulated mRNA expression levels of both *Nrf2* and *Keap1* in the liver and intestine of rice field eels, with consistent trends observed in both tissues. This aligns with the findings of Itoh et al. [43], who demonstrated that Keap1 directs Nrf2 for lysosomal degradation and observed significant increases in relative expression levels of both *Keap1* and *Nrf2*.

### 4.6. Intestinal Histomorphology

The intestine is a vital digestive organ in living organisms. Damage to the morphology of intestinal villi and mucosa can compromise the intestinal barrier, leading to immune-related diseases and impaired growth in fish. VH, VW, and MT are important histomorphological indicators of intestinal development and its digestive and absorptive functions [44]. In this experiment, supplementation with diselenopropionic acid and diselenodibutyric acid significantly increased VH, whereas diselenobutyric acid and diselenodibutyric acid significantly increased VW. Khalil et al. reported that dietary supplementation with different levels of selenium yeast in Atlantic croaker juveniles significantly improved intestinal health, as evidenced by increases in villus length, width, and absorptive area [3]. These results are consistent with the findings of this study. Additionally, the optimal dietary selenium concentration for improving intestinal morphology varies among fish species, likely due to differences in feeding habits, physiological characteristics, farming systems, and feed processing methods. In this experiment, supplementation with diselenobutyric acid and diselenodibutyric acid resulted in fewer intestinal villi and caused villus damage in rice field eels. These compounds may activate pathways such as NF-κB and the NLRP3 inflammasome, leading to the release of inflammatory factors such as IL-1β and TNF-α, which trigger chronic intestinal mucosal inflammation and ultimately result in villus atrophy. Further studies are needed to confirm these mechanisms.

### 4.7. Intestinal Digestive Enzyme Activities

Digestive enzyme activity is a key determinant of a fish’s ability to utilize dietary nutrients and directly influences growth [45]. This study demonstrated that the addition of different organic selenium significantly enhanced the intestinal lipase activity in rice field eels, while it markedly reduced trypsin activity in the Se1, Se2, and Se4 groups and had minimal effect on amylase activity. Dietary supplementation with 0.5 mg/kg and 1.0 mg/kg selenium-enriched yeast in *Apostichopus japonicus* significantly increased the activities of protease, lipase, and amylase in the digestive tract [46]. Similar findings were reported by Li et al., who showed that 0.8 mg/kg selenium-enriched yeast increased digestive tract protease and amylase activity in *Apostichopus japonicus* [47]. The results of this experiment are largely consistent with the above findings, except that selenium supplementation did not affect intestinal amylase activities in rice field eels. This discrepancy may be due to differences in selenium dosage, feeding habits, physiological characteristics, aquaculture practices, or feed processing methods.

### 4.8. Intestinal Microbial Flora

The gut microbiota is a complex microbial ecosystem composed of different bacterial species that interact with each other and play key roles in maintaining host growth and development, resisting pathogenic invasion, and regulating physiological functions [44].

The results of this study have indicated that different forms of organic selenium did not significantly affect the ACE, Chao, Shannon, and Simpson index, which differs from the findings in juvenile turbot (*Scophthalmus maximus* L.) [48]. This discrepancy may be attributed to differences in species, feed composition, and aquaculture environments. Numerous studies have shown that at the phylum level, the dominant gut microbiota in fish primarily consists of *Proteobacteria*, *Fusobacteria*, *Firmicutes*, *Bacteroidetes*, and *Actinobacteria* [49,50]. Among these, *Proteobacteria* is the most predominant phylum, and some of its members are closely associated with host digestive functions. *Proteobacteria* can produce polyhydroxybutyrate as an energy source for intestinal epithelial cells, thereby supporting gut health and promoting fish growth [51]. In this study, both the experimental and control groups were dominated by *Proteobacteria*, *Fusobacteria*, *Firmicutes*, *Bacteroidetes*, and *Actinobacteria*, with *Proteobacteria* being the most prevalent. Other studies have reported that dietary sodium selenite supplementation in juvenile turbot resulted in *Firmicutes*, *Proteobacteria*, *Bacteroidetes*, and *Actinobacteria* as the dominant gut microbiota, with *Firmicutes* showing the highest abundance among all groups [48]. The present findings are consistent with previous studies, indicating that selenium supplementation can maintain the stability of beneficial gut microbiota in fish. However, in this study, supplementation with diselenobutyric acid and diselenodibutyric acid decreased the relative abundance of *Firmicutes* in the gut. This reduction may be associated with decreased ecological requirements for *Firmicutes* resulting from elevated digestive enzyme activities, leading to a lower relative abundance [52]. At the genus level, the abundance of *Cetobacterium* was significantly higher in groups supplemented with diselenoacetic acid and diselenopropionic acid compared to the control and other experimental groups. *Cetobacterium* is commonly found in the intestines of freshwater fish and facilitates the breakdown of peptides and carbohydrates, producing vitamin B12 [53]. Overall, these results suggest that selenium-supplemented feed modulates the intestinal microbiota of rice field eels by decreasing the abundance of potentially harmful bacteria and increasing beneficial bacteria. This modulation may reduce disease incidence and enhance growth performance in rice field eels.

## 5. Conclusions

In summary, dietary supplementation with diselenoacetic acid demonstrates a significant growth-promoting effect in rice field eel. Supplementation with diselenodibutyric acid improves the antioxidant capacity of the liver and intestine, thereby enhancing stress resistance. Dietary supplementation with diselenopropionic acid increases intestinal digestive enzyme activity, preserves villus structure integrity, and increases the relative abundance of beneficial bacteria, thereby enhancing nutrient digestion and absorption in rice field eel. This study presents novel compound feeds and provides important insights for promoting the growth of rice field eel, reducing oxidative stress, decreasing mortality rates, and advancing rice field eel aquaculture.

## Figures and Tables

**Table 1:** Composition and nutrient levels of the basal diet (%).

Ingredients	Content (%)
Fish meal	49.0
Soy protein concentrate	17.0
Corn gluten meal	11.9
Soy lecithin	2.0
Wheat meal	15.0
Fish oil	2.0
Choline chloride	0.5
Ca(H_2_PO_4_)_2_	1.5
Mineral premix ^2^	0.5
Vitamin premix ^1^	0.5
Cellulose powder	0.1
Total	100
Nutrient level	
Moisture (%)	9.82
Crude protein (%)	47.47
Crude lipid (%)	17.67
Crude ash (%)	13.46

Note: ^1^ Vitamin premix (per/kg): VA, 4500 IU; VD3, 1000 IU; VE, 200 mg; VK, 40 mg; VC, 25 mg; VB1, 28 mg; VB2, 80 mg; VB6, 40 mg; VB12, 0.01 mg; VB5, 80 mg; VB7, 0.2 mg; VB9, 4 mg; VB8, 80 mg; and Choline, 500 mg. ^2^ Mineral premix (per/kg): NaCl, 1000 mg; CuSO_4_·5H_2_O, 3.9 mg; FeSO_4_·7H_2_O, 180 mg; ZnSO_4_·7H_2_O, 70 mg; MnSO_4_·H_2_O, 28 mg; MgSO_4_·7H_2_O, 50 mg; CoCl_2_, 0.89 mg; and KI, 0.8 mg.

**Table 2:** Composition and nutrient levels of the basal diet (%).

Gene Name	Primer Sequence (5′–3′)	Product Length (bp)
RPL17	F: GTTGTAGCGACGGAAAGGGAC R: GACTAAATCATGCAAGTCGAGGG	160
CAT	F: GTCCAAGTCTAAGGCATCTCC R: CTCCTCTTCGTTCAGCACC	106
GPX8	F: GTCCACTTACGGTGTTACCT R: ATGGGCTCGTCAGTTCTC	180
Keap1	F: AGCCTGGGTGCGATACGA R: CAAGAAATGACTTTGGTGGG	198
Nrf2	F: CTTCAGACAGCGGTGACAGG R: GCCTCATTCAGTTGGTGCTT	260

**Table 3:** Scheme of all animals and experimental timelines.

Experiment	Time
Aquaculture Experiment	20 July 2024–20 September 2024
Sample Collection	22 September 2024–24 September 2024
Nutritional Components	29 September 2024–30 September 2024
Liver and Intestinal Biochemical Indices	8 October 2024–5 November 2024
Related Antioxidant Gene Expression	10 November 2024–5 December 2024

**Table 4:** Effects of different forms of organic selenium on growth performance in rice field eel (*n* = 3).

Parameters	Se0	Se1	Se2	Se3	Se4
IW (g)	18.53 ± 0.18	18.33 ± 0.00	18.40 ± 0.18	18.40 ± 0.12	18.49 ± 0.08
FCR	0.88 ± 0.18	0.99 ± 0.02	1.07 ± 0.04	1.05 ± 0.15	1.04 ± 0.02
FW (g)	61.93 ± 5.63 ^a^	67.99 ± 5.84 ^b^	62.74 ± 4.41 ^a^	61.94 ± 1.55 ^a^	57.63 ± 6.25 ^a^
WGR (%)	234.02 ± 27.60 ^a^	270.84 ± 31.87 ^b^	240.85 ± 20.65 ^a^	236.65 ± 9.50 ^a^	211.80 ± 35.06 ^a^
SGR (%·d^−1^)	2.01 ± 0.13 ^a^	2.18 ± 0.15 ^b^	2.04 ± 0.10 ^a^	2.02 ± 0.05 ^a^	1.89 ± 0.19 ^a^

Note: Values in the same column with different letter superscripts indicate a significant difference (*p* < 0.05), while those with no or the same small letter superscripts mean no significant difference (*p* > 0.05). The same applies below.

**Table 5:** Effects of different forms of organic selenium on morphological parameters in rice field eel (*n* = 3).

Parameters	Se0	Se1	Se2	Se3	Se4
CF (g/cm^3^)	0.09 ± 0.01	0.10 ± 0.01	0.10 ± 0.01	0.10 ± 0.01	0.09 ± 0.01
HSI (%)	4.64 ± 0.64	4.23 ± 0.17	5.24 ± 0.77	4.38 ± 0.70	4.72 ± 0.22
VSI (%)	7.78 ± 0.65	8.10 ± 0.50	8.99 ± 0.81	8.03 ± 0.33	8.72 ± 0.44

**Table 6:** Effects of different forms of organic selenium on whole-body composition in rice field eel (*n* = 3). Note: Values in the same column with different letter superscripts indicate a significant difference (*p* < 0.05), while those with no or the same small letter superscripts mean no significant difference (*p* > 0.05).

Parameters	Se0	Se1	Se2	Se3	Se4
Moisture (%)	68.04 ± 0.23	66.42 ± 0.90	66.13 ± 0.91	69.99 ± 0.20	68.48 ± 0.02
Crude protein (%)	52.46 ± 1.13 ^b^	50.21 ± 0.88 ^a^	49.74 ± 0.46 ^a^	57.79 ± 0.09 ^c^	51.78±0.96 ^ab^
Crude lipid (%)	34.36 ± 0.12 ^d^	31.38 ± 0.50 ^c^	34.50 ± 0.74 ^d^	26.59 ± 0.22 ^b^	24.62 ± 0.08 ^a^
Ash (%)	7.18 ± 0.69 ^a^	6.35 ± 0.08 ^a^	6.25 ± 0.78 ^a^	8.74 ± 0.56 ^b^	8.88 ± 0.64 ^b^

**Table 7:** Effects of different forms of organic selenium on serum biochemical parameters in rice field eel (*n* = 3). Note: Values in the same column with different letter superscripts indicate a significant difference (*p* < 0.05), while those with no or the same small letter superscripts mean no significant difference (*p* > 0.05).

Parameters	Se0	Se1	Se2	Se3	Se4
TP (g/L)	39.62 ± 3.42	43.22 ± 4.24	46.22 ± 2.69	41.42 ± 2.05	43.65 ± 0.34
ALB (g/L)	13.06 ± 0.47	13.33 ± 1.01	14.20 ± 0.57	12.97 ± 0.36	13.67 ± 0.33
AST (U/L)	63.50 ± 0.50 ^b^	57.00 ± 1.00 ^a^	53.33 ± 1.15 ^a^	93.00 ± 4.36 ^c^	52.50 ± 0.50 ^a^
ALT (U/L)	5.00 ± 1.00 ^bc^	5.33 ± 1.53 ^bc^	4.67 ± 1.53 ^bc^	7.00 ± 1.00 ^c^	2.00 ± 0.00 ^a^
TC (mmol/L)	3.46 ± 0.35 ^a^	3.50 ± 0.45 ^a^	4.39 ± 0.23 ^b^	3.65 ± 0.28 ^a^	4.19 ± 0.20 ^ab^
TG (mmol/L)	1.27 ± 0.28	1.28 ± 0.36	1.55 ± 0.17	1.19 ± 0.27	1.26 ± 0.02
GLU (mmol/L)	4.03 ± 0.49 ^ab^	5.27 ± 0.50 ^b^	4.87 ± 0.95 ^b^	3.60 ± 0.00 ^a^	3.50 ± 0.30 ^a^
HDL (mmol/L)	1.64 ± 0.14	1.65 ± 0.25	2.05 ± 0.06	1.69 ± 0.17	1.57 ± 0.69
LDL (mmol/L)	0.54 ± 0.08	0.49 ± 0.09	0.72 ± 0.07	0.51 ± 0.06	0.51 ± 0.24

**Table 8:** Effects of different forms of organic selenium on the alpha diversity of the intestinal microbial flora in rice field eel (*n* = 3).

Parameters	Se0	Se1	Se2	Se3	Se4
ACE	199.26 ± 18.02	227.04 ± 82.67	168.79 ± 43.76	191.27 ± 14.60	178.84 ± 31.90
Chao	198.87 ± 17.73	229.49 ± 79.70	167.19 ± 46.35	191.14 ± 14.79	177.43 ± 32.02
Shannon	2.98 ± 0.35	2.61 ± 0.91	2.67 ± 0.62	2.96 ± 0.35	2.70 ± 0.46
Simpson	0.16 ± 0.10	0.21 ± 0.13	0.18 ± 0.10	0.16 ± 0.07	0.20 ± 0.09

## Data Availability

The data that support the findings of this study are available from the corresponding author upon reasonable request.
